# Business Modeling to Implement an eHealth Portal for Infection Control: A Reflection on Co-Creation With Stakeholders

**DOI:** 10.2196/resprot.4519

**Published:** 2015-08-13

**Authors:** Maarten van Limburg, Jobke Wentzel, Robbert Sanderman, Lisette van Gemert-Pijnen

**Affiliations:** ^1^ Center for eHealth and Wellbeing Research Department of Psychology, Health and Technology, Faculty of Behavioral, Management and Social Sciences University of Twente Enschede Netherlands; ^2^ Department of Psychology University of Groningen University Medical Center Groningen Groningen Netherlands; ^3^ Department of Medical Microbiology University of Groningen University Medical Center Groningen Groningen Netherlands

**Keywords:** business modeling, co-creation, eHealth, guideline, implementation, road map, stakeholder, value

## Abstract

**Background:**

It is acknowledged that the success and uptake of eHealth improve with the involvement of users and stakeholders to make technology reflect their needs. Involving stakeholders in implementation research is thus a crucial element in developing eHealth technology. Business modeling is an approach to guide implementation research for eHealth. Stakeholders are involved in business modeling by identifying relevant stakeholders, conducting value co-creation dialogs, and co-creating a business model. Because implementation activities are often underestimated as a crucial step while developing eHealth, comprehensive and applicable approaches geared toward business modeling in eHealth are scarce.

**Objective:**

This paper demonstrates the potential of several stakeholder-oriented analysis methods and their practical application was demonstrated using Infectionmanager as an example case. In this paper, we aim to demonstrate how business modeling, with the focus on stakeholder involvement, is used to co-create an eHealth implementation.

**Methods:**

We divided business modeling in 4 main research steps. As part of stakeholder identification, we performed literature scans, expert recommendations, and snowball sampling (Step 1). For stakeholder analyzes, we performed “basic stakeholder analysis,” stakeholder salience, and ranking/analytic hierarchy process (Step 2). For value co-creation dialogs, we performed a process analysis and stakeholder interviews based on the business model canvas (Step 3). Finally, for business model generation, we combined all findings into the business model canvas (Step 4).

**Results:**

Based on the applied methods, we synthesized a step-by-step guide for business modeling with stakeholder-oriented analysis methods that we consider suitable for implementing eHealth.

**Conclusions:**

The step-by-step guide for business modeling with stakeholder involvement enables eHealth researchers to apply a systematic and multidisciplinary, co-creative approach for implementing eHealth. Business modeling becomes an active part in the entire development process of eHealth and starts an early focus on implementation, in which stakeholders help to co-create the basis necessary for a satisfying success and uptake of the eHealth technology.

## Introduction

### Implementation of eHealth

Implementation is necessary to promote the systematic uptake of research findings and other evidence-based practices into routine practice and to improve the quality and effectiveness of health services and care [1]. Attention for evaluating the implementation of eHealth has steadily grown in the last 5 years [2]. Despite this increased attention for implementation, little attention has been given to effects on roles and responsibilities, risk management, engagement of professionals, and transparency of potential benefits of eHealth [2]. Therefore, many implementations are not complete enough when technology “goes live” and its anticipated success is rather a lottery than an actually preplanned implementation. In fact, Black et al [3] concluded in their systematic review that many eHealth projects provide little evidence for actually improving outcomes or being cost effective. Implementation of eHealth has almost universally proven to be more complex and time consuming than anticipated [3]. In addition, many eHealth researchers assume that implementation is an ex-post activity and start preparing implementation when a technology is nearly finished [4]. Many eHealth projects suffer from the “field of dreams” syndrome with the expectation that users will show up as soon as the technology is made available, yet end up having little support, no plans for sustainability, poor uptake, and unknown added value to stakeholders [4,5]. The implementation should not be treated as an afterward necessity, nor treated subordinately to the design of eHealth technology. “Innovation is not just about technology anymore” [6], and therefore, a well-prepared implementation is just as important as a well-designed eHealth technology.

### Business Modeling

In a previous viewpoint paper, we had introduced business modeling as a possible approach to guide the development and implementation of eHealth [4]. Business modeling fosters a ground for dialog regarding the perceived added value and purpose of an eHealth technology [7]. The resulting business model depicts how an organization creates, delivers, and captures value [8]. Such a model can be used as a narrative to explain new ideas [9]. With business modeling, we use this narrative to discuss, plan, and operationalize an implementation of eHealth. Using stakeholder identification, stakeholder analysis, and value co-creation dialog, relevant values can be discussed and then modeled as a business model.

**Figure 1:**
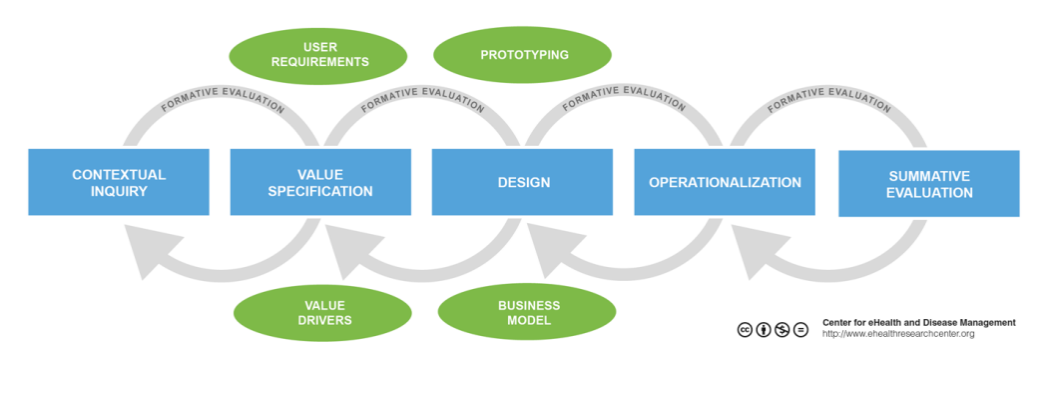
Center for eHealth Research road map.

The Center for eHealth Research (CeHRes) road map (Figure 1) introduces eHealth development as a holistic approach integrating eHealth technology design with business modeling for implementation [4]. The road map consists of the following 5 phases: contextual inquiry, value specification, design, operationalization, and summative evaluation. The road map advises research activities that support eHealth research in each of these phases. This paper expands on this road map by demonstrating the research activities that we apply for business modeling.

### Stakeholder Involvement

Coiera [10] stressed the importance of sociotechnical design in health care. In his paper, he claimed that instead of technology, the social system surrounding that technology should be the central focus. Attention to sociotechnical factors is important to maximize the likelihood of successful implementation and adoption [3]. Academic interest in stakeholder theory started in the late 1970s in the fields of public policy making and business management. The most acknowledged definition for a “stakeholder” in stakeholder theory was established by Freeman as “everyone who affects or is affected by—in this case—the eHealth technology” [11]. A stakeholder analysis aims to evaluate and understand stakeholders from the perspective of an organization to determine their relevance to a project or policy [12]. In 2004, Bryson [13] reviewed 15 stakeholder methods to identify and analyze stakeholders. Although this review described step-by-step instructions for analysis techniques, these techniques focus strongly on expert-driven stakeholder classification without “true involvement” of stakeholders. To sum up, there is adequate information on expert-based stakeholder identification, yet methods or ideas on “how” to involve stakeholders (eg, users, developers, suppliers) as active partakers in stakeholder analysis and further co-creation are less established. Likewise, in implementation research for eHealth, the involvement of stakeholders is still relatively unexplored.

### Aim of This Paper

This paper presents an approach for implementing eHealth with a strong accent on stakeholder involvement. We demonstrate our business modeling research and stakeholder-centered analysis methods in an example case, its added value to implementing eHealth, and conclude with a step-by-step guideline for stakeholder-centered business modeling for eHealth technology.

## Methods

### Stakeholder-Centered Analysis Methods

In a learning-by-doing approach to form our business modeling research, we applied various stakeholder-centered analysis methods in an example case study with a strong focus on discovering how stakeholders can best be involved in business modeling. These stakeholder-centered analysis methods are based on stakeholder theory, existing business modeling tools, and paradigms from human-centered design. In the “Methods” section, we present a theoretical overview for each stakeholder-centered analysis method followed by a practical application as an example and reflections on their application.

### Example Case: Infectionmanager

**Figure 2:**
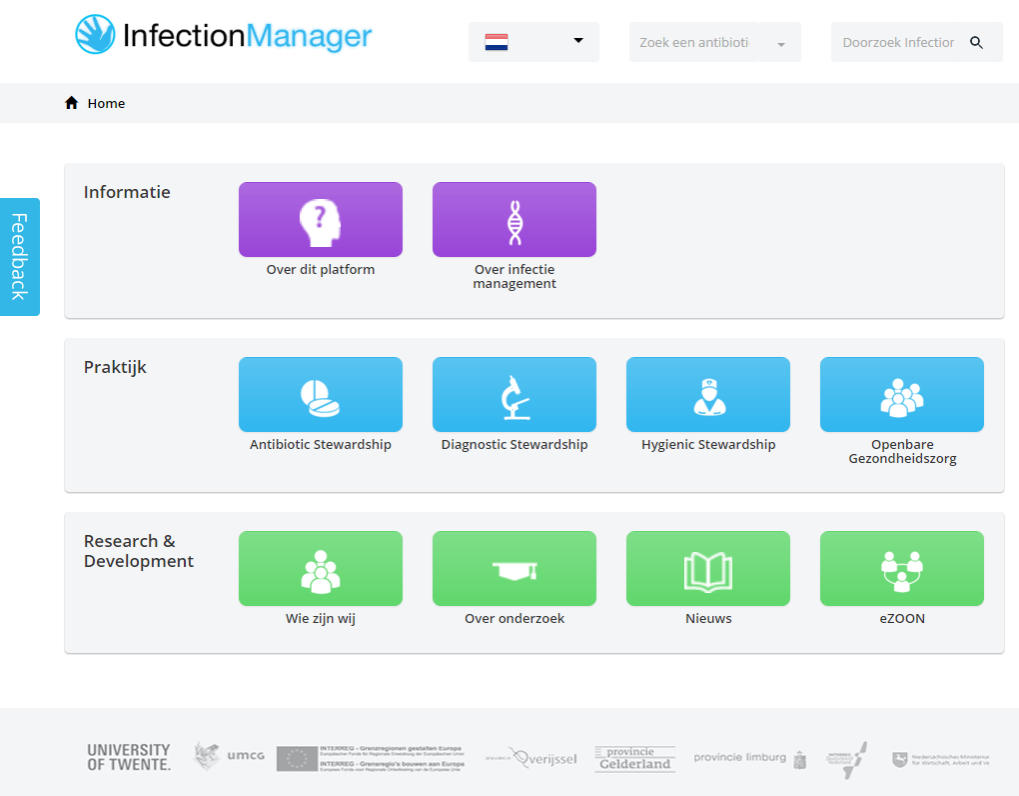
Homepage of Infectionmanager.

The European Union stimulates the mobility of their citizens. Similarly, in health care an increasing number of patients and health care professionals cross the borders and seek or offer health care services abroad. “EurSafety Health-net” has the primary goal to address patient safety in a cross-border context. The EurSafety Health-net consists of 5 “Euregios” or 38 geographical regions, totaling 19.2 million citizens. In these regions, 32 public health organizations and over 300 hospitals participate in the project. Our involvement in this project focuses on developing an Internet-based platform for cross-border infection prevention and control, called “Infectionmanager” (Figure 2). Infection prevention and control is a broad field, and therefore, our eHealth project mainly focuses on antibiotic prescription in hospitals. A change in prescription behavior is urgent, as up to 30-50% of the prescribed antibiotics are either inappropriate or even unnecessary and thereby harming the effectiveness of these antibiotics [14]. Intervening antibiotic use with antibiotic stewardship (ASP) interventions can be a step in curbing antibiotic resistance and hospital-acquired infections, and these can subsequently improve patient safety and reduce costs [15].

The Infectionmanager website is a platform designed to offer eHealth applications that support multiple crucial steps in the antibiotic therapy process and targets multiple, different users and stakeholders. The platform offers eHealth applications with information, decision support, and an overview of the ongoing research and development concerning the platform [16-18]. It targets stakeholders in infection control with currently a specific focus on stakeholders who deal with ASP in hospitals.

The Infectionmanager case is an example of a typical complex eHealth project. First, there is a multitude of stakeholders with diverse stakes, and therefore, an excellent opportunity to try methods for stakeholder involvement. Second, the development options were unlimited, allowing very open discussions with stakeholders to co-create possible eHealth applications and ideas for an implementation. Lastly, the complexity is influenced further by the novelty of ASP in the Netherlands and the novelty of exploring possible eHealth opportunities. Infectionmanager has been researched and developed according to the CeHRes road map [4].

### Stakeholder-Centered Analysis Methods

Involvement of stakeholders changes over time in the research process. In the beginning of an eHealth project, the analysis focuses on finding the right stakeholders and discussing global problems and opportunities, whereas in the later stages of the project, certain opportunities are combined into a possible eHealth technology and the implementation research moves on to value co-creation with topics that deal with added value, feasibility, sustainability, and costs-benefit issues.

In this section, we present each stakeholder-centered analysis method as listed below. First, we give a short summary of the theoretical background of used methods, followed by the practical application in our example case. We conclude each method with some gaps and lessons learned from use and experience.

### Stakeholder Identification

Every eHealth project will have its own unique stakeholder landscape that needs to be understood [4]. As a first step, before analysis *of* or *with* stakeholders can take place, all relevant stakeholders need to be identified. We noticed that stakeholder analysis methods focus more on classification and categorization than identification. Identifying a complete list of the right stakeholders is very crucial for all further analysis. Therefore, the identification step is very important and it is remarkable that it is not described in depth. Many authors consider stakeholders as a default product of a nonexplained identification process [19].

We explored the following 3 approaches to identify stakeholders in an eHealth project: a literature scan/review, expert recommendations, and snowball sampling of stakeholders. These methods are not mutually exclusive and should be integrated as a mixed-method approach for optimal results.

### Stakeholder Identification Method Number 1: Literature Scan/Review

#### In Theory

There are 2 ways to identify stakeholders with literature:

- Identify stakeholders in stakeholder theory. This can result in a list of general types of stakeholders or stakeholders specifically in relation to eHealth.
- Identify stakeholders mentioned in literature on similar (eHealth) interventions.

Ballejos and Montagna [19] recommend starting with identifying stakeholder types [19]. These types of stakeholders can be very diverse, depending on the desired level of detail. Table 1 lists some literature examples from stakeholder’s theory of possible different stakeholders types that can be relevant for eHealth research [19-26]:

**Table 1:** Overview of stakeholder types in literature related to eHealth.

Study	Research focus/setting	Identified stakeholder types
Volere template [24]	Stakeholder roles in information technology	Clients, customers, business/subject experts, future idea specialists, current system specialists, clerical users, technical users, potential users, sales specialists, marketing specialists, aesthetics specialists, graphics specialists, usability specialists, safety specialists, security specialists, cultural specialists, legal specialists, environmental specialists, maintenance specialists, packaging designers, manufacturers, product installers
Wolper [26]	Stakeholders in a typical, large hospital	Competitors, related health care organizations, government regulatory/licensing agencies, private accreditation associations, professional associations, unions, patients, third-party payers, hospital suppliers, media, financial community, special interest groups, religious organizations, local community, nonmanagement medical staff, hospital board, parent companies/organizations, stockholders/taxpayers/contributors, management
Sharp et al [25]	Baseline stakeholders in requirements engineering	Users, developers, regulators, decision makers (with possible client, supplier, and satellite stakeholders for each of the above baseline stakeholders)
Alexander [20]	Product-centric onion model	Developer, maintenance operator, operational support, normal operator, interfacing systems, sponsor or champion, functional beneficiary, purchaser, consultant, political beneficiary, financial beneficiary, negative stakeholders, regulators, the public
Mantzana et al [22]	Health care actors involved in the adoption of information systems	Acceptors, providers, supporters, and controllers
Mettler et al [23]	A total of 4 key stakeholder types with subtypes for eHealth	Service customer, payer of service, responsible for referral, competitor, supplier of goods, supplier of services, supplier of information, government, and community
Ballejos and Montagna [19]	Stakeholder roles (internal or external)	Beneficiaries (functional, financial, political, sponsors), negatives, responsibles, decision makers, regulators, operators, experts, consultants, developers
Hyder et al [21]	A total of 11 stakeholder categories in health care	Beneficiaries, central government agencies, Ministry of Health, local governments, financiers, civil society organizations, health governing boards, provider organizations, professional organizations and health workers, unions, suppliers

Table 1 demonstrates that the stakeholder types can differ for each chosen focus and that multiple focuses can be used to be thorough in the stakeholder identification. Still, these stakeholders are only stakeholder *types*, and therefore, a researcher still has to identify which of these stakeholder types are present and more importantly, identify who the exact stakeholders are for each stakeholder type. For example, relevant stakeholder types can be “users” or “service customers,” but are they patients or specialists? What kind of patients? Which of these patients are included in research and which ones are not?

The second option is to identify stakeholders in the literature on similar interventions. These interventions do not have to be technology per se but are implemented in the same domains as the intended eHealth technology. In this case, very precisely defined stakeholders can be found by looking at the context [27]. This requires sufficient prior knowledge of the domains (medicine, policies, technological) and a clear idea of the goals of the intended eHealth technology. Literature can then be reviewed for mentioned stakeholders (usually professions or organizations); for example, by ranking their occurrence in each publication.

#### Example Case

When starting with Infectionmanager, our research team decided that ASP was a key intervention for infection control in hospitals and that our main interest was to start exploring eHealth possibilities. We conducted a quick scan literature review on ASP to list possible stakeholders who are relevant for ASP [28]. We performed a quick scan (so not a systematic review or similar strict methods) as this list would provide a general idea of stakeholders who should be involved in our ASP research. We ran a query on “antibiotic stewardship” and selected papers of most cited or key literature from that research domain. We scanned 12 key papers and noted every mentioned stakeholder in these papers. This resulted in a complete list of stakeholders in international hospitals based on the literature scan of ASP.

**List of antibiotic stewardship stakeholders identified in a hospital after a literature scan.**

- (Clinical) pharmacists
- Epidemiologists
- Head of pharmacy department
- Infection control nurses
- Infectious disease specialists
- Investigators
- Medical executives
- Medical students
- Microbiologists
- Nurse practitioners
- Nurses
- Pharmacologist experts
- Physicians
- Psychologists
- Software engineers

#### Gaps/Lessons Learned

- A (quick) literature scan is a good starting point to start with stakeholder identification. It is a fast way to draft a list of stakeholders who may be relevant for further stakeholder identification and later stakeholder analysis.
- An inventory of stakeholder types can be useful as an extra check to see if certain stakeholder types are missing on the stakeholder list or left out for a clear reason.
- Start with a manageable amount of key publications using a simple query in your research subject and list or tally mentioned stakeholders. With 10-20 publications, that stakeholders list will saturate.
- New, innovative health care interventions have limited available literature, especially in an academic context. In our example case, little literature was available for eHealth/health care technology in the field of prescribing antibiotics and stewardship.
- A potential danger with international literature is that it describes various different health care contexts and thus identified stakeholders may not be relevant for local health care systems. To illustrate with examples from our project: Microbiologists in Francophonic countries are called “bacteriologists,” and thus, are not 2 different stakeholders; or “infectious disease physicians” do not exist as-is in the Dutch health care system and the closest comparable profession would be an “infectologist,” which we learnt afterward through validation of our stakeholder list with experts.
- Policies, (clinical) protocols, and documents are very relevant sources to take into consideration as literature for stakeholder analysis [29], especially when the eHealth intervention is targeted toward supporting tasks performed by health care professionals. Obtaining these protocols and documents requires access via experts or stakeholders who use them.

### Stakeholder Identification Method Number 2: Expert Recommendations

#### In Theory

After exploring stakeholders from a theoretical perspective, the next step is to introduce a practical perspective. Most stakeholder analysis methodologies seem to prefer an expert-driven approach. According to Bryson [13], the “basic stakeholder analysis technique” suggests that the planning team (ie, the eHealth research team in eHealth context) brainstorms which stakeholders should be included for analysis. Depending on the composition of the planning team, one can also ask (external) field experts to nominate stakeholders [21]. The goal of this brainstorming session is to make a complete overview of relevant stakeholders to the eHealth project.

#### Example Case

**Figure 3:**
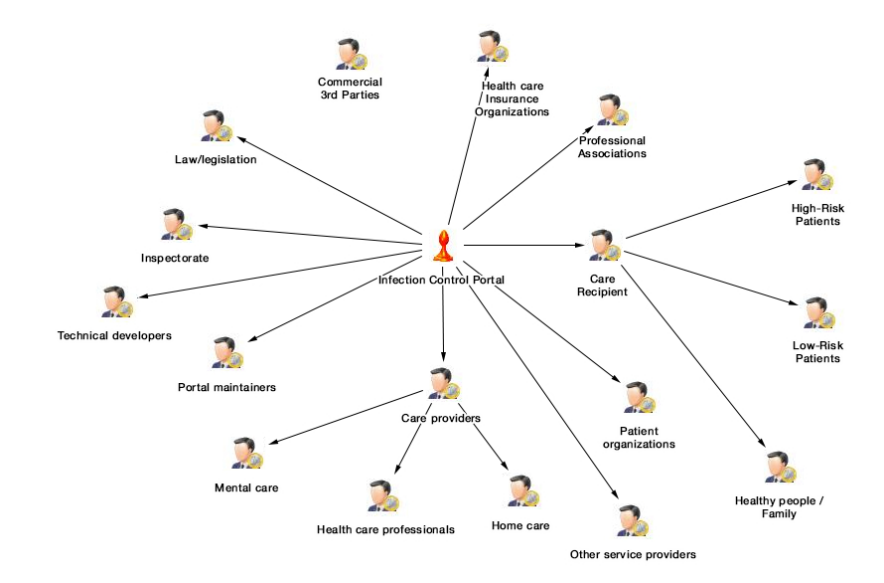
Stakeholder map relevant for infection control and subsequently Infectionmanager.

We planned 2 brainstorming rounds. The first round started by using specific software that allowed to visualize stakeholder mapping. Our planning team consisted of eHealth researchers and infection control experts affiliated with our EurSafety Health-net project. We conducted 22-hour brainstorming sessions to visualize an overview of stakeholders relevant for infection control and subsequently Infectionmanager. In this early phase of our research, we looked at infection management, which had a broader scope than ASP specifically. We also categorized the stakeholders in stakeholder groups with the mapping software. The Infectionmanager was the central point of discussion, and so, the central question was “Which people or organizations have an influence on Infectionmanager?” And subsequently, “Which people or organizations are influenced by Infectionmanager?.” Using these 2 questions, we brainstormed a stakeholder map. In this visual way of brainstorming, the network and relationships of stakeholders become clear. For example, the stakeholder “care recipients” can be categorized into 3 different types of care recipients with different roles toward Infectionmanager. Or, as another example, we listed possible commercial third parties, possible hospitals, and so on. The visualization aspect of this approach helps to draft a visual representation of the possible stakeholder map, which makes the brainstorming process less abstract and more comprehensive for all participants in the brainstorming team. A global overview of our stakeholder map can be seen in Figure 3.

The second brainstorming round targeted “ASP” more specifically and was a continuation of the stakeholders found with the quick literature scan as described in the previous method. Our team of eHealth researchers asked an infection control expert working at a pilot hospital to help us transpose the theoretical list of (international) stakeholders to stakeholders present at a pulmonary ward. We chose this pulmonary ward, as these wards have a relatively high use of antibiotics and relatively low multimorbidity. In the focus group, we brainstormed about every stakeholder on the list and the stakeholder’s possible role in ASP, Dutch analogous profession, and whether that stakeholder was available in the pulmonary ward. Later, for further stakeholder analysis, we organized a focus group with the following stakeholders [30]: clinical microbiologists, pharmacists, (chest) physicians, residents, nurses, nurse manager, ward manager, and staff members of management.

#### Gaps/Lessons Learned

- Brainstorming with experts is a useful method to bridge the theoretical list of stakeholders with the relevant practice. Experts are active in the field, so a researcher needs to make use of their firsthand knowledge.
- Visualization of the stakeholder map helps making the discussion of relevant stakeholders less abstract and fosters the collaboration and discussion. A map is quick to comprehend and easier to share than long lists, for example. It also visually structures the mentioned stakeholders.
- The more experts involved, the better. Experts are limited to their profession and background and may not know all parts of the stakeholder map. For example, a microbiologist knows all about the laboratory and microbiological diagnosis but has little insight into the daily routine of a nurse during ASP.
- Structure in the focus group is important. Prevent vociferous stakeholders who hijack the session for sharing their views only. Give every stakeholder adequate time and attention.
- Involving more experts also increases validation and paints a broader picture.
- Be open-minded to the stakeholders that experts suggest despite prior knowledge from the literature. In case of questionable or unclear stakeholders, note them and ask why they are relevant and discuss/evaluate their relevance later with other experts.

### Stakeholder Identification Method Number 3: Snowball Sampling With Stakeholders

#### In Theory

Both literature and expert recommendations can still miss certain stakeholders who may be important to the project. A final step, once a list of stakeholders is ready, is to ask these stakeholders to complete the list. The added value of this step is to validate the list of stakeholders from a stakeholder’s perspective and a last chance to identify missing stakeholders. Snowball sampling is a technique where existing participants recruit future participants among their acquaintances. In terms of stakeholder snowball sampling, stakeholders can be asked who the stakeholders are, or, in case of an already available list, which stakeholders are important and which ones are missing. Snowball sampling is one of the common methods used for stakeholder identification [31].

#### Example Case

**Figure 4:**
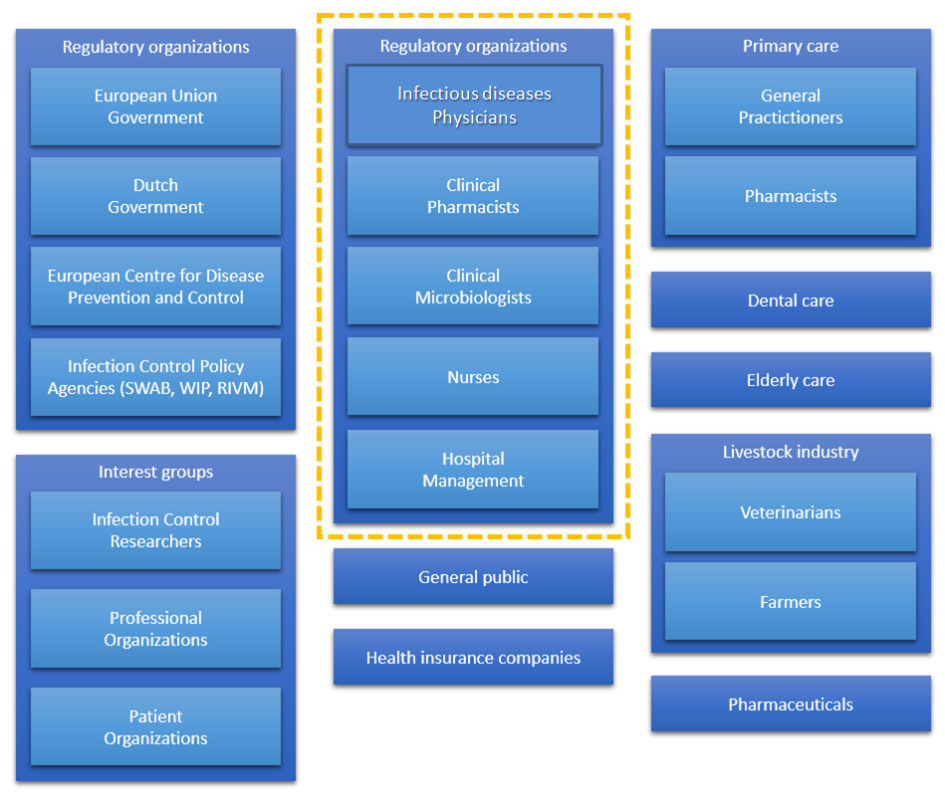
Example of all the stakeholders relevant for infection control.

In earlier brainstorm sessions (mentioned as a “previous method”), we drafted an initial list of stakeholders in infection control. These stakeholders were sent a questionnaire in which they could rank the importance of stakeholders on our stakeholders list and suggest missing stakeholders [32]. This eventually resulted in the stakeholder map of infection control as depicted in Figure 4. What is interesting is that this map contains some different stakeholders than the ones mentioned by experts and us but above all, it has a broader focus than the expert-based map in Figure 3. For example, our research mostly focused on stakeholders related to infection control in hospitals; yet, these stakeholders also pointed out that dental care and livestock industry deal with infections and antibiotics and are very relevant for infection control as a whole. Therefore, despite having a focus on hospitals (as outlined in Figure 3), there are a lot more other infection control stakeholders to involve in the stakeholder analysis.

As mentioned in the “Stakeholder Identification Method Number 2: Expert Recommendations” section, we further focused on ASP as a key intervention for infection control inside hospitals. Thus, we planned a focus group with stakeholders at a pulmonary ward [30].We also applied snowball sampling to this focus group, and the existing stakeholders agreed that we should additionally contact dieticians, cleaning personnel, and a representative of the information technology department as they may have an influence on ASP.

#### Gaps/Lessons Learned

- Stakeholders have the most direct firsthand experience within the subject domain and thus are crucial in stakeholder identification.
- Snowball sampling is suitable for identifying missing stakeholders. In our case, for example, we identified neither dieticians nor cleaning personnel as relevant stakeholders for ASP via literature.
- Questionnaires are the most convenient method for snowball sampling a complete list.
- Focus groups allow interaction with stakeholders, to iteratively assess conclusions from stakeholders and researchers. Yet, focus groups can be difficult to organize, especially when they consist of a high number of stakeholders. The focus group needs to have something for the stakeholders to be willing to schedule it.

#### Stakeholder Analysis

After stakeholders are identified, they can take part in the stakeholder analysis. Not every identified stakeholder will be equally important to the implementation of the eHealth technology [4]. In addition, it takes time and resources to interact with every single stakeholder, and therefore, it is recommendable to work toward a selection of key stakeholders. Narrowing the list of stakeholders requires applying some acceptable and justifiable sorting criteria [33]. Again returning to the review by Bryson [13], there are a plethora of stakeholder analysis methodologies to classify stakeholders. In this paper, we demonstrate our application of the basic stakeholder analysis, stakeholder salience (Mitchell’s classification), and ranking/analytic hierarchy process (AHP) method.

### Stakeholder Analysis Method Number 1: Basic Stakeholder Analysis

#### In Theory

The basic stakeholder analysis method involves brainstorming expert-based opinions on behalf of each stakeholder [13]. The research team and/or experts can give a global impression from the stakeholders’ point of view about what the expectations can be for each possible stakeholder. The analysis aspect behind this method is that a stakeholder with many (important) expectations will most likely be important to the project throughout development. In terms of business modeling, these expectations are related to “values” that we will discuss more in depth later in this paper.

Looking from a research team stance, this overview of possible expectations also allows a first impression on the value proposition possibilities [34]. A value proposition is “the value created for users by the offering based on technology” [35]. In other words, it describes what added value a technology has to offer, as well as possible services around the technology. This value proposition will be the basis for the design and implementation of the eHealth technology.

#### Example Case

During our brainstorming sessions early on in our research, we examined with experts what possible values each stakeholder could express. We used the same stakeholder mapping software by Inpaqt again to make a value tree for every stakeholder. Value trees can be used to identify a hierarchy of values [36]. For each stakeholder, our project team discussed possible value expectations of Infectionmanager. The next step was to assign a level of importance to these value expectations. We assigned a number between 1 and 5 for each value and its attributes. Not only can this method prioritize stakeholders with many (high-ranking) value expectations, but it can also provide an overview of possible value needs and how these values and their attributes are linked with each other. In this example, providing information for high-risk patients with the Infectionmanager (the attribute “information”) would not only affect the value “be informed” but also the values “feeling better,” “empowerment,” and “peace of mind”.

**Figure 5:**
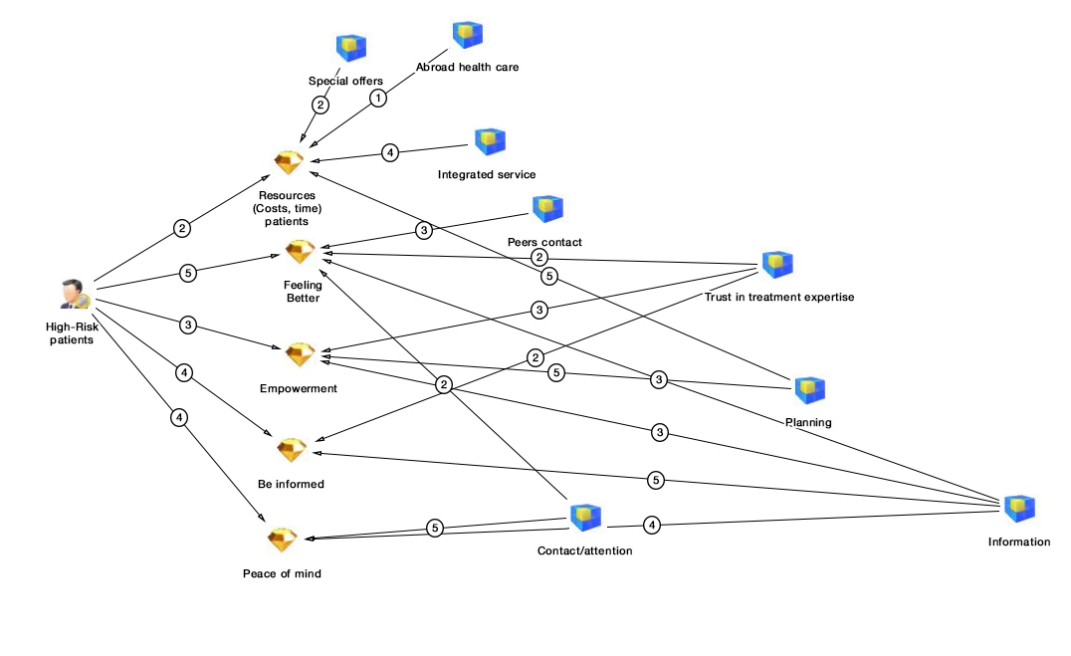
Example of value tree with possible values expectations and attributes for high-risk patients.

In Figure 5, we show an example of a value tree with possible values (diamonds)—expectations of a high-risk patient group—as well as attributes (blue boxes) that detail these values.

#### Gaps/Lessons Learned

- This method is a start to understand who the possible important stakeholders are and to prepare a general impression on what to expect as value needs for the technology and implementation.
- It helps to understand the linkage of values. For example, the same values can be shared by multiple stakeholders or values can influence the technology on several places and vice versa.
- Theoretically, this “basic stakeholder analysis” method does not truly involve stakeholders because it is done by experts. To make this method less expert driven and more stakeholder driven, stakeholders can partake in the stakeholder analysis sessions as well.
- Doing this digitally can be a bit more difficult as during the brainstorming sessions a researcher has to real-time model while conducting the discussions, although this is very convenient for continuing and sharing the session results.
- The analysis remains subjective and rather high level or abstract as you try to draw an overall picture of all possible views of all possible stakeholders with experts.
- Experts only see their part of the process, and thus, their conceived values may be biased.

### Stakeholder Analysis Method Number 2: Stakeholder Salience

#### In Theory

**Figure 6:**
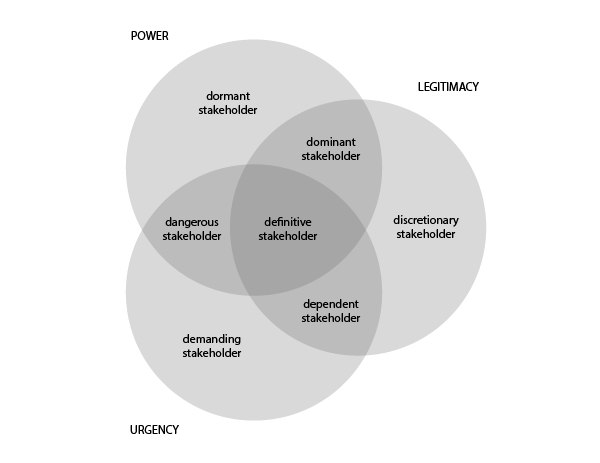
Stakeholder salience diagram according to Mitchell et al [34].

A popular method to determine the importance of stakeholders is the stakeholder salience approach proposed by Mitchell et al [33]. They defined stakeholder salience as the degree to which managers give priority to competing stakeholder claims. Salience is based on 3 attributes that can be classified, namely, power, legitimacy, and urgency (Figure 6). *Power* is defined as “a relationship among social actors in which one social actor, A, can get another social actor, B, to do something that B would not have otherwise done.” *Legitimacy* is defined as “a generalized perception or assumption that the actions of an entity are desirable, proper, or appropriate within some socially constructed system of norms, values, beliefs, and definitions.” Finally, *urgency* is defined as “the degree to which stakeholder claims call for immediate attention.” Based on the 3 attributes, Mitchell et al [33] defined 9 possible stakeholder classes for classification. It is out of the scope of this paper to elaborate on each class, but in short, stakeholders who score on all 3 attributes are definite stakeholders, and thus key stakeholders. Stakeholders who score 2 of 3 are relatively dominant, dependent, or dangerous stakeholders and should also be included. Stakeholders who only score 1 of 3 are dormant, discretionary, or demanding stakeholders.

Stakeholder salience can be determined by experts in the aforementioned expert brainstorm sessions or project meetings, or by stakeholders themselves using a questionnaire, one-on-one interviews, or a focus group.

#### Example Case

We first arranged expert interviews to rate the infection control stakeholders, according to Mitchell’s salience. We asked 2 infection control experts to rate stakeholders on whether they have power, legitimacy, and urgency. We then sent a questionnaire to stakeholders who according to experts were the “definite stakeholders” [32]. Table 2 shows a fragment of our salience assessment.

Practically, we learnt that these 3 attributes of salience (ie, power, urgency, and legitimacy) are difficult and had to be explained in more general, nonbusiness-specific terms to the experts and stakeholders: We explained power as “the level of influence a stakeholder has in infection control.” Legitimacy was explained as “the level in which a stakeholder needs to be legally, morally, or contractually involved in infection control.” And finally, urgency was “the priority or necessity of the stakeholder in infection control.” It is crucial to keep these terminologies and definitions consistent [31].

After comparing the stakeholder salience expressed by stakeholders and by experts, we could validate and draw consensus in both results [32]. The differences were that experts mentioned the Ministry of Health as important and stakeholders did not, and stakeholders found the National Institute for Public Health and the Environment, nurses, and veterinarians more salient. We added these 3 to our final definite stakeholders list.

**Table 2:** Example of a classification of infection control stakeholders using Mitchell’s stakeholder salience.

Stakeholder	Power	Legitimacy	Urgency	Type
Medical specialist/physician	X	X	X	Definite
General practitioner (GP)	X	X	X	Definite
GP assistant	—	X	X	Dependent
Clinical microbiologist	X	X	X	Definite
Nurse	—	X	X	Dependent
Pharmacist	X	X	X	Definite
National Institute for Public Health and the Environment	—	—	X	Demanding
Dutch Working Group on Antibiotic Policy	X	X	X	Definite
Medicines Evaluation Board	X	X	—	Dominant
Insurance companies	X	—	—	Dormant

#### Gaps/Lessons Learned

- This salience approach is the most commonly used method to assess the importance of stakeholders, and thus, can be seen as a widely acknowledged method. It is also a commonly used method for stakeholder assessment in eHealth research.
- Determining which stakeholders are definite stakeholders—in-turn important for implementation research—is feasible using Mitchell’s stakeholder salience. This is especially true when it is necessary to bring the number of stakeholders down to a manageable number to actively involve them in the implementation research.
- The 3 salience attributes (ie, power, legitimacy, and urgency) are difficult concepts. They might overlap and as they are explained in business terms, they are also complex to properly explain to stakeholders. The researchers have to be consistent in the explanation and make sure the stakeholders understand the difference.
- Subsequently, there is also a risk that stakeholders do not fully comprehend the attributes and give answers based on gut feelings or what they expect should answer. Therefore, as the researcher, one needs to be alert and ask for short elaborations.
- The stakeholders who score all 3 attributes of salience are important stakeholders to be involved in the project; however, with a high number of stakeholders, it is important that further analysis is carried out to identify those stakeholders who scored 2 (or maybe even 1) of 3 attributes and include them in the list. This depends on the number of stakeholders and keeping it manageable for research purposes.

### Stakeholder Analysis Method Number 3: Ranking/AHP

#### In Theory

Another way to classify the importance of stakeholders is by attributing an importance score to stakeholders. This scoring or ranking can be done in several ways. In our research, we used a 5-point scale and a derivative of AHP [37] as 2 methods for ranking:

- The 5-point scale is very straightforward. Hyder et al [21] proposed to articulate the power or importance of stakeholders using a 5-point scale. Experts or stakeholders themselves can assign 0 (not important) to 5 (very important) points to a list of stakeholders. Similar methods can deviate from the scale, eg, a 9-point scale [36] but different scales seem arbitrary.
- A mathematically more sophisticated method for ranking is Saaty’s AHP [37], which is also applied in health care research [38]. It is out of the scope of this paper to explain how AHP works in full detail. In short, AHP is frequently used in the analysis for decision making. In AHP, the hierarchic relation (an eigenvector approach) of stakeholders weights their relative importance. Saaty’s AHP technique becomes especially interesting when the hierarchy expands by also mapping values and attributes to stakeholders (as seen in the value trees in Figure 5). Using a mathematical construction, the number of values and hierarchic relationships determine a weighted outcome for every stakeholder, value, and attribute. It is a sophisticated method, but in our experience the most thorough analysis currently available.

#### Example Case

**Figure 7:**
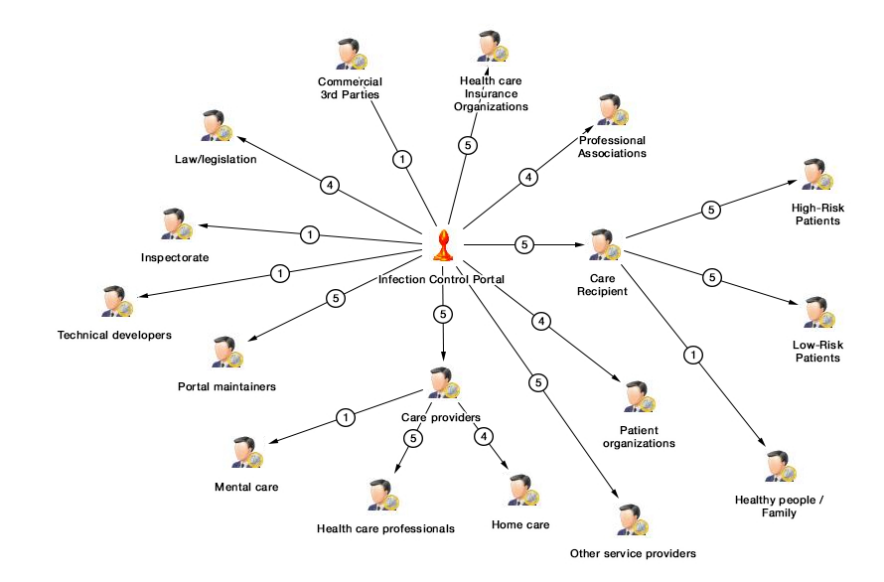
Expert-based stakeholder ranking for infection control portal.

The software tool we used for ranking the stakeholders also allowed for a 0-5-point scale to rate the importance of stakeholders. We applied a simple hierarchic calculus based on the value trees. For example, a value with 5 points from a stakeholder with 5 points would get 25 points, a value with 5 points from a stakeholder with 2 points would score 10 points. This is slightly different to Saaty’s AHP method as we did not apply relative weights and eigenvectors to avoid overcomplexity in the calculations. We assigned the ranking in a brainstorm session with experts as can be seen in Figure 7 and we did the same to values (as already shown in Figure 5). We did not rank stakeholder or values in an interactive session with stakeholders themselves in our example case, as it would be unfeasible to organize all stakeholders together.

#### Gaps/Lessons Learned

- Ranking with numbers is a simple yet effective way to quantify and classify the importance of stakeholders.
- AHP can overkill and in practice a simpler calculation of [stakeholder× value × attribute] might be a good alternative.
- The 0-5-point scale is still an arbitrary quantification that is interpreted by stakeholders or experts. For example, what makes a stakeholder a 2 or a 3? The best way to get satisfying results is by validation by either asking multiple stakeholders to rank or to work toward a consensus.
- We did not choose to fully use AHP because it has to be done very thorough, as the hierarchy will determine the importance through eigenvectors. If 1 stakeholder or value is lacking, results may become counterintuitive [37]. More research is needed on this.

### Value Co-Creation Dialog

After the stakeholders are analyzed and it is known whose input to the implementation of the eHealth technology is more important than others, it is time to start with value co-creation. We define a “value” as an ideal or interest a (future) end user or stakeholder aspires to or has [29]. These values can be further detailed into “attributes.” An attribute is a summary of the need or wish that is spoken out by the (future) end user or stakeholder [29]. Still, “value” remains a difficult concept to concisely communicate as this can elicit philosophical debates on what is good and bad. The eventual eHealth technology and its surrounding services to embed it properly in its intended care setting all encompass the value of the eHealth technology.

Value co-creation is a joint activity involving customers to identify values from their perspective [39]. In other words, with co-creation, stakeholders get an active dialog and co-design the development process of eHealth. In addition, for most stakeholders, value is also a difficult business concept to grasp. One cannot simply ask, “Ok, what value do you expect?” In fact, in most cases the stakeholders cannot even grasp what the technology will be like, nor how it can be used. The researcher has to prepare relevant value co-creation questions and have a discussion with all key stakeholders about their value expectations.

We herein demonstrate 2 possibilities as to how we conducted these value co-creation dialogs: process analysis and stakeholder interviews using the business model canvas.

### Value Co-Creation Dialogs Method Number 1: Process Analysis

#### In Theory

To co-create value, Prahalad and Ramaswamy [39] noted that a joint problem definition and problem solving are required. To facilitate this process, the authors recommend the DART method:

- have “dialogs” with stakeholders about their experiences;
- get “access” to information;
- assess “risks” and benefits with stakeholders; and
- be “transparent” with information.

We combined these 4 with ideas of the contextual inquiry of our road map that recommends performing interviews or focus groups using a scenario-based problem analysis. Focus groups offer an opportunity to obtain insights regarding the experiences, observations, and opinions of group members [40]. As Prahalad and Ramaswamy [39] point out, to understand the individual experiences for co-creation, the problem analysis, inspired by action research, sense making [41], and previous research [42], should encompass a general discussion of the entire process, including individual tasks, information, and communication needs, as well as the problems experienced and bottlenecks.

#### Example Case

We organized a workshop for a focus group in a pulmonary ward, inviting stakeholders relevant for ASP [30]. In this workshop, we asked stakeholders about the problems they experienced (general), process bottlenecks (coordination, communication), and information needs (communication, documentation). Stakeholder role playing (enact a situation or process) is mentioned as a possible way to determine importance and value needs of stakeholders [43]. Thus, we started a quick role play of “Who does what?” with the process behind antibiotic prescription for a complex patient. For each topic, we prepared a poster on which stakeholders could stick written Post-its with possible values, and group them in importance. The main problems that were mentioned were regarding the information flow of patient information and insufficient cooperation and consultation between the attending physician and microbiologists again due to inefficient information sharing as well as due to unstructured procedures for consultation. Some stakeholders also noted that an insufficient knowledge of (new) procedures or application of medication might cause problems [30]. An interesting find was that nurses could play a big role in ASP.

#### Gaps/Lessons Learned

- Value creation with a focus group approach allows for a discussion, and therefore, when talking about processes, problems, or tasks, stakeholders can directly respond to each other, allowing co-creation through agreement and consensus on possible positive and negative values.
- This discussion itself can already be an eye-opening experience for stakeholders. On several occasions, a stakeholder admitted, “I did not know that you were experiencing that (as a) problem,” which suggests that discussions create more understanding for each other and willingness for improvement or change.
- Stakeholders might not express all problems or play them down due to the presence of other stakeholders.
- Through this approach, stakeholders will mostly discuss problems and opportunities to change these problems. They might not express them exactly as values but more as attributes. In that case, after recording the focus group sessions, researchers need to extract values from the transcript that are relevant to the technology and its implementation [29].
- In this step also eHealth opportunities can be discussed that can help ideating possible eHealth technology in collaboration with the (technical) design researchers.

### Value Co-Creation Dialogs Method Number 2: Business Model Building Blocks

#### In Theory

For this approach, we started with a business model as a basis to discuss values. A business model mediates between technology development and its intended (economic) value creation [6,35]. In other words, it can be used to explain the value creation logic necessary to create a successful piece of technology. Likewise, a business model can explain the rationale behind implementing eHealth technology [4]. The most commonly used framework for making a business model is the business model canvas by Osterwalder and Pigneur [8]. Their business model consists of the following 9 building blocks: value propositions, customer relationships, channels, customers, key activities, key resources, key partners, cost structure, and revenue streams. These building blocks can guide questions regarding the necessary values for implementing eHealth. Although Osterwalder and Pigneur [8] proposed several questions for each building block, these are targeted toward high-level strategic management. The trick is to transpose these questions to the intended eHealth technology and ask which values are necessary for that eHealth technology to be successful.

#### Example Case

We took the building blocks of the business model canvas and organized them into 4 main topics for questions on necessary values for implementing ASP, taking the mentioned problems and bottlenecks during the focus group into consideration when preparing questions. Table 3 presents some questions used. Each of the 4 topics has a central question that needs to be answered, with several subquestions. We then organized 1-hour, one-on-one interviews with stakeholders and used this questionnaire as a basis for the interview.

**Table 3:** Example of topics based on business model components.

Building blocks	Central question	Explanation
Value proposition (the technology and its services)	What value should antibiotic stewardship (ASP) offer?	The value proposition is basically the to-be-developed platform for ASP. We prepared concrete questions like “What value does ASP need to deliver to you, to your department, and to the hospital?,” “What problems does it help to solve?,” “What technology and services can we offer to you?,” and “What do you deem really necessary to be satisfied with ASP?”
Customers, key resources, and key partners (the stakeholders)	Who are the stakeholders?	Here we focused on all human interactions relevant for ASP. We asked which stakeholders (people or organizations the stakeholder interacted with, or should interact with for ASP). We made a list of stakeholders, described their role briefly, and ranked their importance. We also asked for external stakeholders who may be relevant for ASP as, in general, stakeholders tended to respond from their internal, hospital perspective.
Key resources and key activities (the infrastructure)	What is the required infrastructure?	We asked “How can ASP be integrated with your daily routine?” Regarding possible resources, we asked what tools, means, documents, sources, or people were necessary for ASP and their importance. We had to steer the stakeholder by asking specifically whether a certain technical infrastructure is needed, what technical medium, which data flows and connections or systems are relevant to assess the needs for eHealth technology. We also steered by asking what knowledge is further required, in terms of support from people or literature to have an ASP to assess what resources are specific to ASP.
Costs and revenues (the added values)	What are the success factors?	We avoided monetary discussions with stakeholders. Costs and revenues are always a difficult subject as there may be many benefits not directly linkable to 1 particular stakeholder. In the focus group we organized earlier, stakeholders stated there is a trade-off between quality and efficiency regarding ASP and that they should be balanced [30]. Therefore, we chose to ask for effects and success factors. We asked what the expected effects on patient outcomes (eg, length of stay, mortality, treatment duration, patient safety) would be and their relative importance and whether other quality aspects not directly related to the patient are relevant. We did the same for efficiency, and so, what are the important outcomes for efficiency (costs, less usage of antibiotics, fewer complications, etc) and their importance.

#### Gaps/Lessons Learned

- One-on-one interviews allow for in-depth analysis of possible values and critical success factors for implementing an eHealth technology and results in a deeper discussion and understanding of each stakeholder’s value expectations. Not only are values expressed, but it is also elaborated why they are important.
- From our experience, we advise that the questions need to be concrete enough for stakeholders to give satisfying answers. If the questions are too abstract, the answers will be equally abstract and thus less useful.
- It is important that the interviewer focuses on what the technology should contribute, not design or requirements. It is not about how they want the eHealth solution to be, it is about the why.

### Business Model Generation

As “business modeling” suggests, the eventual output is a business model. Exact visualizations of business models are diverse and there is no unanimous agreement on what they exactly should look like or the level of detail they should contain [7]. This is also why there is neither a dominant design nor many tools available for making business models. A popular method for visualizing a business model is the business model canvas [8]. Although this canvas is perfect at abstracting and visualizing key elements that should be *in* a business model, comprehensive step-by-step instructions on *how* to retrieve the detailed narrative for these key elements remain rather abstract and is therefore mostly targeted at high-level strategic management. However, existing templates or blueprints such as this business model canvas are useful to make a model representation of an implementation of health care technology [4]. We also used this business model canvas as our template for a business model.

### Business Model Generation Method: Business Model Canvas

#### In Theory

**Figure 8:**
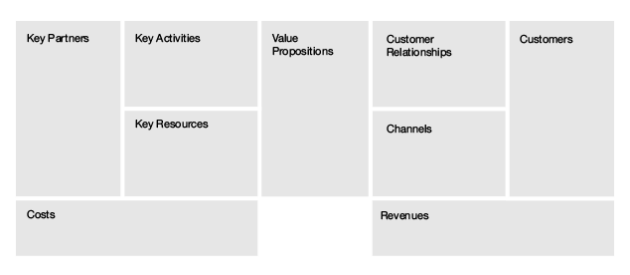
Business model canvas by Osterwalder and Pigneur [8].

The business model canvas (Figure 8) consists of 9 building blocks that can describe the whole rationale of an implementation. In the middle block is the value proposition, the eHealth technology in this case. The top 3 blocks on the left-hand side of the model deal with the required organizational and infrastructural aspects, that is, the key activities, resources, and partners. The top 3 blocks on the right-hand side deal with who the customers/users are and how to interact with them. At the bottom are the financial aspects. Creating and offering values generate costs, and a revenue model is necessary to capture value back to at least cover these costs. This canvas can be used as an empty framework or blueprint to fill with critical success factors that describe the implementation of an eHealth technology.

#### Example Case

**Figure 9:**
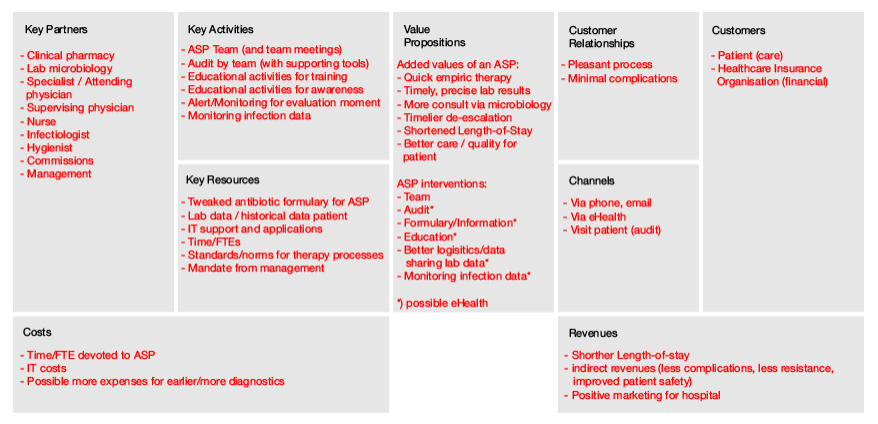
Business model canvas filled with critical success factors.

We made a business model (Figure 9) filled with the values that were concluded using the focus group and one-on-one interviews as delineated in the previously explained value co-creation methods. We listed critical success factors that are our translation of expressed values and attributes.

This business model in Figure 9 gives an overview of relevant critical success factors that determine the success of ASP and what role Infectionmanager can play in ASP. It pinpoints critical values that the technology needs to offer to be valuable to stakeholders, critical values that need to be made available in the infrastructure to guarantee feasibility, uptake, and sustainability. This business model also gives an idea about financial opportunities that are available to make Infectionmanager self-sustainable. To sum it up, this business model provides a bird’s-eye view of all critical success factors to implement our Infectionmanager.

#### Gaps/Lessons Learned

- A business model can give an overview of the critical success factors for implementing an eHealth technology.
- The level of detail depends on the dialogs with stakeholders, and therefore, the completeness of the business model depends on the (successful) completion of those earlier research steps.
- This is still only a model that reflects a possible (maybe even multiple) implementation. It still needs to be explained to others and practically expanded on to put the eHealth technology “live.”

## Results

### Stakeholder Analysis and Co-Creation of Values

Stakeholder analysis and co-creating values for a business model with them is a progressive journey to understand the global context and problems and to gradually work toward an in-depth, individual dialog with stakeholders to understand what they find important to the technology and its implementation. By exploring several stakeholder-oriented methods as part of business modeling as delineated in the “Methods” section, should we have to start implementation research anew from scratch, we would suggest the business modeling steps presented in Textbox 2.

**Step-by-step guideline for stakeholder involvement for business modeling in eHealth technology implementation.**

Business modeling steps for implementing eHealth technology (arranged stepwise):

1. Start with a literature review on comparable interventions to get a feeling for the domains, jargon, and global issues and stakeholders.
2. Involve 1 or 2 domain experts in the research and development team to reflect future findings, ideally experts who have an affinity with technology and research processes.
3. Make an overview of all possible stakeholders based on literature on comparable interventions in the domain.
4. Assign stakeholder types to possible stakeholders, verify if certain types are missing and why.
5. Validate the entire overview by snowball sampling a complete stakeholder list with these key stakeholders.
6. Let experts select key stakeholders from the complete stakeholder list.
7. Organize a focus group with at least one in-person representative of each key stakeholder:
  - Start with discussing each stakeholders’ role in the current processes.
  - Let them complete the stakeholder list for missing stakeholders based on the process.
  - Ask stakeholders to rank the importance of stakeholders, or alternatively let experts do it later.
  - Discuss what bottlenecks are experienced.
  - Discuss opportunities for improvement and opportunities for eHealth.
8. Summarize bottlenecks and opportunities and determine with the research team which opportunities are there for eHealth technology and whether these fit the project goals.
9. Ideate an eHealth technology (when possible, make mock-ups or a prototype of the ideas).
10. Plan interviews with stakeholders, or if possible, multiple stakeholders of the same stakeholder type, for value co-creation dialogs for the ideated eHealth technology.
11. Prepare the value co-creation dialog interview with questions that address all business model components (also prepare subquestions that propose possible ideas or values on each business model component to help the interview along. Focus on what the technology should contribute to their daily routines, not technical requirements).
12. Code transcripts of the focus groups and interviews, extract all implementation-related comments and combine all values and critical factors in the business model canvas.
13. Discuss the resultant business model with the research team.
14. Optionally, for transparency and extra validation, explain the business model to stakeholders and let them reflect on it or write a document that explains the implementation strategy based on the business model as the model itself may be unclear to share with the relevant stakeholders.

### Gaps/Lessons Learned

To further substantiate the guideline, we conclude the following main lessons from the gaps and lessons learned from our implementation research, for which the aforementioned guideline will help:

- Understanding the context beforehand is crucial to find the right stakeholders and to understand their problems and opportunities for eHealth. As an eHealth researcher, you will have to familiarize yourself with the relevant domains. In our example case, we read up on antibiotics and microbiology literature. If the domain is not your core expertise, involving an expert from the domain is a must to help validating the research.
- Identifying stake*holders* is easier than identifying their *stakes*. Stakeholder analysis is a complex task and needs to be done thoroughly to understand which stakeholders play a key role in the implementation of eHealth technology. Our advice is to discuss it with a group of stakeholder or combine multiple analyses so that outcomes can be compared.
- Co-creation requires incorporating multiple perspectives. Eventually, everything is joined in an implementation. When important stakeholders have different or even incompatible views on the implementation, this will become a huge problem for the technology. All effort should then go toward finding a consensus or a workable trade-off between values.
- Values are tough constructs. Business modeling is about discussing values, but stakeholders usually do not express their views in terms of “greater goods,” but in to-the-point, pragmatic statements of what they want or what should be changed. It is up to the research team to interpret and combine these statements into high-level values.
- Business models are not all about money. Health care is a complex market in which, for example, quality of care or patient safety can be much more important than cost savings or maximized profits. Therefore, the values to discuss are truly “greater goods” and not only money flows.
- An implementation is never finished. Every environment is dynamic, so stakeholders change, business models change, technologies change, etc. The technology needs to be evaluated and when outcomes are getting unsatisfactory it may be worthwhile to redo the business modeling steps iteratively to see what has changed and how these changes can be anticipated.

## Discussion

### Preliminary Findings

In this paper, we propose a guideline for business modeling with stakeholder-oriented analysis methods for implementing eHealth. The aim of this guideline is to co-create an implementation for eHealth together with stakeholders, by identifying and analyzing stakeholders, discussing co-creation of value with stakeholders, and determining a business model. Once all values are captured in a business model following the step-by-step guide, the model can be used as a basis to disseminate or further detail the design and implementation of the eHealth technology.

We saw that most applications of business models in eHealth (*if* applied that is) are usually based on generic, strategic models or concocted by experts without truly involving stakeholders in the process. In that regard, there is little to no co-creation with stakeholders. The proposed guideline may seem a lot of research and time consuming, but if it can avoid misaligned plans or expectations, lack of uptake, or even design mistakes, it should be worth to spend that time and effort in business modeling.

Because only few frameworks or guidelines are available for business modeling, we chose a pragmatic approach for determining a guideline that can be used in future implementation research. The CeHRes road map (Figure 1) originated in the search to combine “design research” with “implementation research” for a holistic approach for health care technology development. Design and implementation influence each other; hence, a holistic view that combines both is essential for the success of health care technology [4]. Health care technology development is a multidisciplinary process [44]. However, in the field of health care, a multidisciplinary and participatory approach toward development is novel as many of these projects are still expert or eminence driven. This causes problems, as experts also are biased in how they perceive the setting. Policymakers or management see the big picture and understand the global problems a technology needs to address, but still details necessary for implementation can only be understood by talking to those who are directly influenced by the technology.

Stakeholder analysis theory is less scarce than theory on business modeling. In fact, there are many methods in the academic field such as stakeholder theory, policy making, or requirements engineering. Yet, all these possible methods have to be combined in the context of eHealth development. eHealth brings multiple domains of research together; thus, it calls for experimenting with combinations of multidisciplinary research methods. We believe this guideline is a first step toward a very pragmatic approach to think about an implementation for eHealth technology with the essence that stakeholders should be involved in the entire process.

Whereas other implementation theories such as normalization process theory [45], service, technology, organization, and finance model [46], human, organization and technology-fit [47] focus on advising possible factors that influence eHealth implementation, we focused on obtaining such possible factors from stakeholders themselves. Although the aforementioned methods may be successful to find an implementation, we believe that the focus on stakeholders helps to make the technology fit their daily routines and environment in a bottom-up approach. It basically emulates the principles of user/human-centered design, by co-creating an implementation with stakeholders. Instead of a top-down approach in which experts work with a preset of possible critical factors, we apply a bottom-up approach by extracting possible critical factors from what stakeholders deem critical for implementation.

Considering the difficulties with implementation of eHealth as we laid out in the “Introduction” section, we found that describing a pragmatic approach for co-creating an implementation with stakeholders may spur others to be more transparent in how they did it. Instead of reinventing the wheel or repeating the same mistakes again, eHealth projects can learn from each other by giving more insights into the steps that were taken to implement the technology.

### Limitations

The presented guideline also has some limitations. First, this paper only demonstrated 1 example case. We applied individual methods or parts from the guideline in parallel to eHealth research based on our CeHRes road map [4,42,48-50], yet further validation of its generic use as a complete framework for other eHealth projects is necessary. It is certainly worthwhile to test the guideline in different research settings as well as compare differences in the results of its methods to see what works best under different conditions.

Second, the proposed activities can be very thorough and time consuming. Going through them faster or being less thorough is an option when time or resources are limiting factors. This suggests opportunities for future research to determine which methods are crucial or which can be left out or possible quicker or discount variants on the methods to find a balance between investing minimal time and satisfactory results. For example, it would not make sense to spend a few years researching the possible relevant stakeholders in a quickly changing environment like eHealth and technology.

Finally, this paper was written over time while exploring all instruments for business modeling, and therefore, our choices for these instruments were based on our good and bad experiences and constraints posed by our projects.

### Future Research

We applied the business modeling steps in our example case and also applied them in other projects to test whether they can be used in various projects. In future road map-related publications, we plan to further expand on the business modeling steps and their applications to other eHealth projects. At present, there is 1 eHealth project on zoonoses that is starting with the stakeholder identification and analysis steps. In another eHealth project on dermatology, our business modeling steps are also applied thoroughly and can be published as a second example case.

### Conclusions

A successful, sustainable implementation of eHealth technologies is still a tough nut to crack for many eHealth projects and we believe that more involvement of stakeholders in the whole development process of eHealth, and not only designing the actual technology but also designing its implementation can improve the overall success of the eHealth project. Having a dialog with stakeholders about their value expectations will help researchers and developers—as well as all involved stakeholders—to understand what and why they are developing eHealth technologies. We hope we can spark others to work with our proposed guideline, or try stakeholder involvement and business modeling, to advance research in the implementation of eHealth.

## Abbreviations

AHP: analytic hierarchy process
ASP: antibiotic stewardship (program)
CeHRes: Center for eHealth Research
